# Pelvic floor magnetic stimulation combined with biofeedback-based electrical stimulation for obstructed defecation syndrome: a retrospective cohort study

**DOI:** 10.3389/fmed.2026.1982655

**Published:** 2026-10-29

**Authors:** Kun Tang, Run Zhang, Kui-ling Wang, Rui Ding, Wen-hui Liu, Er-Juan Wang, Ming Li

**Affiliations:** 1 Department of Anorectal Surgery III, The First Affiliated Hospital of Anhui University of Chinese Medicine, Hefei, China; 2 Graduate School, Anhui University of Chinese Medicine, Hefei, China

**Keywords:** biofeedback-based electrical stimulation, constipation, obstructed defecation syndrome, pelvic floor magnetic stimulation, quality of life

## Abstract

**Background:**

Obstructed defecation syndrome (ODS) is a clinical syndrome of impaired rectal evacuation that may involve functional and/or structural anorectal abnormalities. Biofeedback-based electrical stimulation (BF-ES) is commonly used in conservative management, while pelvic floor magnetic stimulation (PFMS) is sometimes added in clinical practice. Comparative evidence for the combined regimen remains limited.

**Methods:**

A retrospective study was conducted in patients with ODS who received systematic treatment at our Pelvic Floor Treatment Center between January 2021 and January 2026. Patients were divided according to their treatment regimen into the BF-ES group and the PFMS plus BF-ES group. A total of 260 patients (*n* = 130 per group) were included. All patients underwent an 8-week standardized rehabilitation intervention. Primary outcomes included weekly complete spontaneous bowel movement (CSBM) responder rate and pelvic-floor surface electromyography (sEMG) parameters. Secondary outcomes included weekly laxative-use days, the Constipation Symptom Score (CSS), Traditional Chinese Medicine (TCM) constipation syndrome score, the Patient Assessment of Constipation Quality of Life questionnaire (PAC-QOL), and adverse-event incidence.

**Results:**

Most baseline covariates met the prespecified balance criterion (SMD < 0.2), although a modest residual imbalance remained in baseline weekly laxative-use days (SMD = 0.243). Compared with BF-ES alone, the PFMS plus BF-ES group had higher CSBM responder and overall response rates (91.5% vs. 80.0% and 97.69% vs. 82.31%, respectively; both *P* < 0.05), with greater improvements in weekly laxative-use days, TCM constipation syndrome score, PAC-QOL, and selected sEMG parameters (all *P* < 0.05). Regression analyses showed that baseline laxative use was associated with greater improvement in the TCM constipation syndrome score improvement, whereas laxative use during treatment was associated with less improvement (both *P* < 0.001). Sex was independently associated with PAC-QOL change (*P* < 0.05).

**Conclusions:**

PFMS plus BF-ES was associated with better clinical outcomes than BF-ES alone in patients with ODS. Prospective randomized controlled trials are warranted to confirm these findings.

## 1 Introduction

Chronic functional constipation is one of the most common disorders of gut–brain interaction and imposes a substantial burden on patients’ daily activities, health-related quality of life, and healthcare resource utilization. Recent Rome V global epidemiological data reported a prevalence of chronic constipation of 8.9% among adults (1). Earlier population-based studies further indicated that constipation is more prevalent among women, older adults, and individuals with lower socioeconomic status (2, 3). Within the spectrum of chronic constipation, obstructed defecation syndrome (ODS) is a clinical syndrome characterized by impaired rectal evacuation. Its underlying abnormalities may be functional, such as pelvic-floor dyssynergia, structural, such as rectocele, rectal or rectoanal intussusception, rectal prolapse, enterocele or pelvic-floor hernia, and excessive perineal descent, or a combination of both. Clinically, patients commonly report excessive straining, a sensation of incomplete evacuation or anorectal blockage, and the need for manual maneuvers to facilitate defecation (4). Among patients presenting to tertiary referral centers for constipation, approximately one-half exhibit features related to obstructed defecation (5). The clinical importance of ODS lies in the fact that persistent difficulty in rectal evacuation is often accompanied by prolonged symptom burden, recurrent laxative use, and substantial impairment in quality of life (6).

Pelvic floor retraining with biofeedback therapy (BFT) is an established treatment for functional defecation disorders, particularly dyssynergic defecation (7, 8). Randomized controlled trials and reviews have reported clinically meaningful improvement in approximately 70% of patients with dyssynergic defecation treated with BFT (7, 8). However, these findings should not be generalized to all patients with ODS because functional and structural anorectal abnormalities may coexist and may respond differently to conservative treatment (6). A recent study of patients with refractory constipation and outlet dysfunction found favorable responses to BFT in patients with dyssynergic defecation and inadequate defecatory propulsion, whereas patients with isolated structural outlet obstruction had substantially lower response rates (9). These findings underscore the heterogeneity of ODS and provide a rationale for evaluating adjunctive treatment strategies in patients with an incomplete response to biofeedback-based therapy.

Adjunctive stimulation-based strategies have increasingly been incorporated into pelvic floor rehabilitation. In patients with pelvic floor dyssynergia, a randomized trial showed that biofeedback combined with transanal electrical stimulation produced sustained improvements in bowel symptoms, anorectal function, and constipation-specific quality of life compared with standard conservative treatment (10). More recently, a randomized controlled trial in women with defecography-confirmed moderate rectocele-associated ODS used transanal electrical stimulation combined with biofeedback as the conventional rehabilitation regimen; adding pelvic floor magnetic stimulation further improved pelvic floor muscle recruitment, anorectal function, and constipation-related quality of life (11). Pelvic floor magnetic stimulation (PFMS) is a noninvasive neuromodulatory technique that induces electrical currents in pelvic tissues through electromagnetic induction and has been investigated across several pelvic floor disorders. Earlier evidence was derived predominantly from urological and urogynecological conditions, particularly urinary incontinence, overactive bladder/detrusor overactivity, and chronic pelvic pain (12, 13). Direct evidence regarding PFMS for defecatory disorders and ODS remains limited. Functional magnetic stimulation improved colonic transit and defecographic parameters in a small prospective study of patients with Parkinson disease–associated constipation (14), while the recent rectocele-associated ODS trial provides more direct but phenotype-specific evidence for PFMS as an adjunct to electrical stimulation and biofeedback (11). Thus, whether adding PFMS to BF-ES provides additional clinical benefit across a broader, clinically heterogeneous ODS population remains uncertain.

Therefore, this retrospective cohort study aimed to compare the clinical efficacy and safety of PFMS plus BF-ES with BF-ES alone in patients with ODS and to provide preliminary evidence for future prospective randomized controlled trials.

## 2 Methods

### 2.1 Study design and study population

This single-center retrospective cohort study included patients with clinically identified ODS. All participants fulfilled the Rome IV diagnostic criteria for functional constipation (15) and had symptoms of impaired rectal evacuation supported by digital rectal examination, pelvic-floor surface electromyography (sEMG), and defecography. All patients received standardized treatment at the Pelvic Floor Treatment Center of a tertiary Grade A hospital in Anhui Province, China. Clinical data were retrieved from the hospital electronic medical record system between January 2021 and January 2026. Patients were categorized according to the treatment they had received in routine clinical practice. Treatment allocation was not randomized. The treatment regimen was determined by the treating clinician after baseline clinical and anorectal assessment, taking into account the patient's clinical presentation, anorectal findings, suitability for PFMS, and patient preference. Patients who received biofeedback therapy combined with electrical stimulation were classified as the BF-ES group, whereas those who additionally received pelvic-floor magnetic stimulation were classified as the PFMS plus BF-ES group. To reduce potential confounding, 1:1 propensity-score matching (PSM) was performed based on age, sex, disease duration, pelvic-floor dyssynergia (absent/present), baseline weekly laxative-use days, and baseline weekly CSBM frequency. After matching, 130 patients were included in each group. Details of the matching procedure are provided in Section 2.7. The study workflow is shown in Figure 1. The study was conducted and reported in accordance with the Strengthening the Reporting of Observational Studies in Epidemiology (STROBE) statement (16).

**Figure 1:**
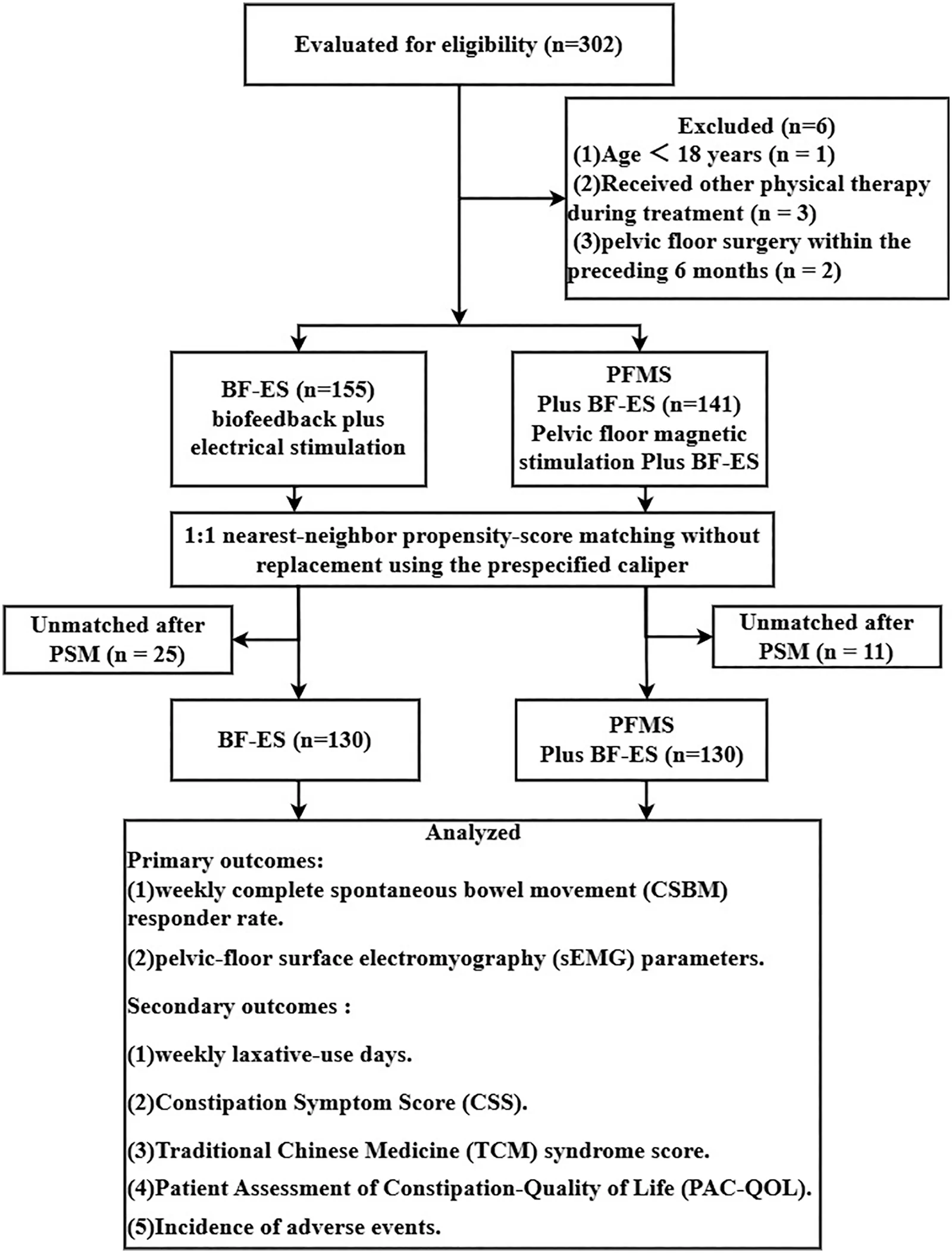
Research flowchart.

### 2.2 Ethical considerations

This study was approved by the Ethics Committee of Anhui University of Chinese Medicine (Approval No.: 2025AH218) and conducted in accordance with the Declaration of Helsinki and international ethical guidelines. Given the retrospective design utilizing archived clinical data, the requirement for informed consent was waived by the Ethics Committee. All patient-related data were de-identified to safeguard participants' privacy throughout the study.

### 2.3 Eligibility and diagnostic assessment

Patients were required to fulfill the Rome IV diagnostic criteria for functional constipation (15). Symptoms had begun at least 6 months before diagnosis, with the diagnostic criteria fulfilled during the preceding 3 months. At least two of the following were required: straining, lumpy or hard stools, a sensation of incomplete evacuation, a sensation of anorectal obstruction or blockage, or manual maneuvers during ≥25% of defecations, or fewer than three spontaneous bowel movements per week. Loose stools were rarely present without laxatives, and the criteria for irritable bowel syndrome were not fulfilled. ODS was clinically identified by prominent symptoms of impaired rectal evacuation supported by digital rectal examination, pelvic-floor sEMG, and defecography.

### 2.4 Inclusion and exclusion criteria

Inclusion criteria were: (1) age 18–85 years; (2) fulfillment of the Rome IV diagnostic criteria for functional constipation, together with symptoms of impaired rectal evacuation and objective anorectal findings consistent with an outlet evacuation disorder on digital rectal examination, pelvic-floor sEMG, and defecography; (3) completion of at least 8 weeks of treatment according to the specified protocol; and (4) availability of complete clinical records required for analysis.

Exclusion criteria: (1) concomitant slow transit constipation; (2) pelvic floor surgery within the preceding 6 months; (3) implantation of an electronic medical device, including a cardiac pacemaker; (4) severe cognitive dysfunction or psychiatric illness that could interfere with treatment or assessment; (5) opioid use within the previous 6 months; (6) concurrent administration of other physical treatments that may impact the evaluation of treatment efficacy during the course of treatment.

### 2.5 Treatment protocols

Before treatment initiation, all patients underwent standardized baseline evaluations, including review of medical history and psychological evaluation. Standardized education was then provided by trained therapists regarding the physiology of normal defecation together with the therapeutic rationale, treatment procedures, and expected goals of PFMS and BF-ES. The specific treatment plans for each group of patients are as follows:

BF-ES: Patients in this group received BF-ES therapy using the Extended-4 system (Vishee Medical Technology Co., Ltd., Nanjing, China), five sessions per week. Before treatment, qualified rehabilitation therapists provided one-on-one instruction on correct pelvic-floor muscle contraction, emphasizing anal sphincter contraction without recruitment of the abdominal, gluteal, or thigh muscles. Treatment was performed in the supine position with the lower limbs comfortably abducted. An anal probe was inserted with the second conductive ring positioned 1 cm proximal to the anal verge. Surface electrodes were placed bilaterally above the anterior superior iliac spines, and an additional pair of abdominal electrodes was positioned approximately 1 cm below the umbilicus. Each 40-min session comprised 20 min of neuromuscular electrical stimulation (NMES) followed by 20 min of sEMG-guided biofeedback training. NMES was delivered through the anal probe using a symmetrical biphasic pulsed current at 30 Hz with a pulse width of 200 μs. Each stimulation cycle consisted of a 1-s ramp-up, 5-s stimulation, 1-s ramp-down, and 5-s rest period. Stimulation intensity was gradually increased from zero to the highest comfortably tolerated level below the pain threshold. During the subsequent biofeedback phase, real-time sEMG activity was visually displayed to guide voluntary contraction and relaxation of the pelvic-floor and anal sphincter muscles. The initial sEMG target was approximately 10 μV and was adjusted to 15–20 μV according to individual performance. These μV values represented the sEMG biofeedback target and not the electrical stimulation output intensity.

PFMS combined with BF-ES: Patients in this group received PFMS in addition to BF-ES. PFMS was delivered using an M-100 Ultimate pulsed magnetic stimulator (Shenzhen Yingchi Technology Co., Ltd., Shenzhen, China) equipped with a dedicated pelvic-floor stimulation coil. The stimulator has a maximum specified magnetic flux density of up to 7T and generates biphasic magnetic pulses. PFMS parameters were selected within the range routinely used in clinical practice and individualized according to patient tolerance and observable pelvic-floor muscle responses. Patients were seated on the treatment chair with the legs naturally separated and the anus positioned directly over the magnetic stimulation coil. Stimulation was delivered at 10–15 Hz, with the stimulator output adjusted to 20%–40% of the maximum stimulator output (MSO). Each stimulation train lasted 5 s, followed by a 5–10-s inter-train interval. The intensity was adjusted to produce perceptible rhythmic pelvic-floor muscle contractions without pain or discomfort. Each PFMS session lasted 20 min and was administered once daily, five sessions per week. PFMS parameters were not assigned according to the presence of structural anorectal abnormalities or pelvic-floor dyssynergia.

### 2.6 Outcome measures

Data for the following observational parameters were retrospectively collected by reviewing hospital-based electronic medical records, communication records, paper-based medical charts and scale questionnaires.

#### 2.6.1 Demographic and clinical characteristics

Baseline characteristics included demographic information (age and sex), disease duration, baseline bowel-function characteristics (weekly laxative-use days and weekly CSBM frequency), previous treatment history, and anorectal structural and functional characteristics. Defecography records were reviewed to identify structural anorectal abnormalities, including rectocele, rectal or rectoanal intussusception, rectal prolapse, enterocele or pelvic-floor hernia, and excessive perineal descent. Pelvic-floor dyssynergia was defined by documented paradoxical contraction or inadequate relaxation of the puborectalis and/or anal sphincter during attempted defecation. Structural anorectal abnormalities and pelvic-floor dyssynergia were recorded independently because they could coexist in the same patient.

Because this was a retrospective study, several clinically relevant variables were incompletely documented in routine practice and were therefore unavailable for analysis. These variables included obstetric history, rectocele grade, severity of enterocele or internal intussusception, balloon expulsion test results, and the degree of pelvic floor descent. Consequently, these factors were not incorporated into the propensity score matching procedure or the multivariable regression analyses.

#### 2.6.2 Weekly CSBM responder rate

CSBM was included as a primary clinical outcome in accordance with the relevant consensus recommendations (17). A spontaneous bowel movement (SBM) was defined as a bowel movement occurring without rescue laxative use during the preceding 24 h, whereas a CSBM referred to an SBM associated with a sensation of complete evacuation. The weekly CSBM responder rate was defined as the proportion of patients who fulfilled the prespecified responder criteria. Post-treatment weekly CSBM frequency was defined as the number of CSBMs recorded during the final treatment week. A weekly CSBM responder was defined as a patient who achieved at least three complete CSBMs within a given week and an increase of at least one CSBM per week from baseline (18). In the present study, this criterion was applied to the final week of the 8-week treatment period (week 8), which served as the post-treatment assessment window.

#### 2.6.3 Pelvic-floor surface electromyography (sEMG)

Pelvic floor function was evaluated by sEMG before and after treatment using a pelvic floor sEMG assessment system (SA9800, Vishee Medical Technology Co., Ltd., Nanjing, China). Outcome measures included the mean resting baseline, coefficient of variation of the resting baseline, relaxation time following rapid contraction, and coefficient of variation during endurance contraction.

#### 2.6.4 Weekly laxative-use days

The number of days on which laxatives were used during the week before treatment and during the final week of treatment was recorded. Changes in weekly laxative-use days were compared between baseline and post-treatment assessments.

#### 2.6.5 Constipation symptom score (CSS)

Constipation severity was evaluated using the CSS, originally developed by Agachan et al. (19). The scale comprises eight items covering key constipation-related symptoms, including bowel movement frequency, straining during defecation, and sensation of incomplete evacuation, with total scores ranging from 0 to 30. Higher scores indicate greater symptom severity.

#### 2.6.6 Traditional Chinese medicine (TCM) constipation syndrome score

The TCM constipation syndrome score (Supplementary Table S1) was developed based on the Expert Consensus for the Integrated Chinese-Western Medicine Diagnosis and Management of Functional Constipation (17). This scale included constipation-related symptoms, systemic accompanying manifestations, and tongue-pulse signs. Each symptom item was graded using a four-level severity scale: none, mild, moderate, and severe. Primary symptoms were scored 0, 2, 4 or 6 points, while secondary symptoms were scored 0, 1, 2 or 3 points. Tongue and pulse findings were classified as normal or abnormal. Abnormal signs were further categorized as severe (2 points) or mild (1 point) according to severity, whereas normal signs were assigned 0 points. Higher total scores represented more severe TCM constipation syndrome score. Clinical efficacy was evaluated using the TCM syndrome efficacy index, calculated as: TCM syndrome efficacy index (%) = [(pre-treatment TCM constipation syndrome score − post-treatment TCM constipation syndrome score)/pre-treatment TCM constipation syndrome score] × 100. According to the predefined efficacy criteria, patients were classified as cured (efficacy index ≥95%), markedly improved (efficacy index ≥70% and <95%), improved (efficacy index ≥30% and <70%), non-responder (efficacy index <30%). The overall response rate was calculated as the proportion of patients classified as cured, markedly improved, or improved, that is, those with an efficacy index ≥30%.

#### 2.6.7 Patient assessment of constipation quality of life questionnaire (PAC-QOL)

Quality of life was assessed using the validated Chinese version of the Patient Assessment of Constipation Quality of Life questionnaire (PAC-QOL) (20, 21). The PAC-QOL consists of 28 items across four domains: physical discomfort, psychosocial discomfort, worries and concerns, and satisfaction. Each item is scored from 0 to 4. In the present study, the overall PAC-QOL score was calculated as the mean of all 28 items, yielding a score ranging from 0 to 4, with higher scores indicating poorer constipation-related quality of life.

#### 2.6.8 Safety outcomes

Adverse events (AEs) occurring during treatment and follow-up were recorded, including local pain, pelvic floor muscle soreness or spasm, skin burning sensation, rectal bleeding, perianal skin injury, and clinically unfavorable increases in bowel movement frequency accompanied by diarrhea and/or fecal urgency. The severity of AEs was graded as mild, moderate, or severe according to the Common Terminology Criteria for Adverse Events (22). Serious adverse events (SAEs) were defined as events requiring hospitalization or additional medical intervention.

### 2.7 Statistical analysis

Statistical analyses were conducted using IBM SPSS Statistics, version 26.0. Due to baseline imbalance between groups resulting from the study design, 1:1 propensity-score matching (PSM) was conducted for the two treatment groups to reduce confounding bias. The propensity-score model included age, sex, disease duration, pelvic-floor dyssynergia (absent/present), baseline weekly laxative-use days, and baseline weekly CSBM frequency. A logistic regression model was used to estimate propensity scores. Patients were matched using a nearest-neighbor matching algorithm with a caliper width of 0.2 times the standard deviation of the logit-transformed propensity score, and matching was performed without replacement. After matching, standardized mean differences (SMDs) were calculated to assess covariate balance between the two groups; an SMD <0.2 was considered indicative of good covariate balance between the two groups. Given the retrospective nature of this study, data completeness was verified. No missing data were found for the primary and secondary outcomes in the final matched sample; therefore, no imputation methods were applied. Continuous variables were summarized as mean (SD) or median (IQR), and categorical variables as *n* (%). Between-group differences were examined using the independent-samples *t* test, Mann–Whitney *U* test, or *χ*^2^ test, as appropriate. Separate multivariable linear regression models were used to assess factors associated with changes in CSS, TCM constipation syndrome, and PAC-QOL scores. Each model included age, disease duration, weekly laxative-use days before and during treatment, sex, structural anorectal abnormality (absent/present), pelvic-floor dyssynergia (absent/present), treatment regimen, laxative use before treatment (no/yes), and laxative use during treatment (no/yes). Multicollinearity among covariates was assessed using variance inflation factors (VIFs) and tolerance values, with VIF <5 and tolerance >0.20 considered indicative of no problematic multicollinearity. Change scores were calculated as post-treatment minus baseline values; therefore, more negative values indicated greater improvement. Clinical efficacy grade was analyzed using a multivariable proportional-odds logistic regression model with the same covariates. The proportional-odds assumption was assessed using the test of parallel lines. A nonsignificant result (*P* > 0.05) was considered to indicate no evidence of violation of the proportional-odds assumption. Efficacy was coded as 0 = non-responder, 1 = improved, 2 = markedly improved, and 3 = cured, with higher values indicating better therapeutic efficacy. Odds ratios (ORs) with 95% confidence intervals (CIs) were reported; an OR >1 indicated greater odds of being classified in a higher, more favorable efficacy category, whereas an OR <1 indicated lower odds. Model-estimated probabilities for each efficacy category were calculated for each patient and averaged within treatment groups. All tests were two-sided, with *P* < 0.05 indicating statistical significance.

## 3 Results

### 3.1 Comparison of baseline characteristics between the two groups

Before propensity score matching, 155 patients were included in the BF-ES group and 141 in the PFMS combined with BF-ES group. Before matching, the two groups were comparable with respect to mean age (52.57 vs. 53.52 years, *P* = 0.58), disease duration (6.07 vs. 6.32 years, *P* = 0.74), and sex distribution (women: 105/155 [67.7%] vs. 95/141 [67.4%], *χ*^2^ = 0.005, *P* = 0.946). However, the prevalence of pelvic-floor dyssynergia differed significantly between the BF-ES and PFMS plus BF-ES groups (64/155 [41.3%] vs. 39/141 [27.7%], *χ*^2^ = 6.046, *P* = 0.014). After 1:1 nearest-neighbor matching, 130 patients remained in each group. Most baseline characteristics were adequately balanced between the two groups (Table 1). Structural anorectal abnormalities were present in 108 patients (83.1%) in the BF-ES group and 109 patients (83.8%) in the PFMS plus BF-ES group (SMD = 0.021). Pelvic-floor dyssynergia was present in 39 patients (30.0%) in each group (SMD = 0.000), indicating excellent post-matching balance for these anorectal characteristics. A modest residual imbalance remained in baseline weekly laxative-use days (SMD = 0.243), although the between-group difference was not statistically significant (*P* = 0.252).

**Table 1:** Baseline characteristics of the propensity score–matched groups.

Variable	BF-ES group (*n* = 130)	PFMS plus BF-ES group (*n* = 130)	*Z*/*χ*^2^	*P* value	Absolute SMD
Age (years), median (IQR)	56.5 (40–62.25)	55 (43.75–63.25)	−0.012	0.99	0.06
Disease duration (years), median (IQR)	4 (2–10)	4 (1–10)	−1.039	0.299	0.02
Days of laxative use per week before treatment, median (IQR)	5 (4–6)	5 (5–6)	−1.146	0.252	0.243
Weekly CSBM frequency before treatment (times/week), median (IQR)	2 (2–3)	2 (1–3)	−0.586	0.558	0.145
Sex, male/female, *n*	42/88	42/88	0	1	0
Structural anorectal abnormality, *n* (%)	108 (83.1)	109 (83.8)	0.028	0.867	0.021
Pelvic-floor dyssynergia, *n* (%)	39 (30.0)	39 (30.0)	0	1	0
Laxative use before treatment, no/yes, *n*	8/122	3/127	–	0.216^a^	0.192

Data are presented as median (IQR), *n*, or *n* (%), as appropriate. Continuous variables were compared by Mann–Whitney *U* test; categorical variables were compared using Pearson *χ*^2^ or Fisher's exact test. Quartiles were calculated with SPSS EXAMINE using the default HAVERAGE percentile method.

a Fisher's exact test.

### 3.2 Comparison of primary efficacy outcomes between the two groups

Baseline weekly CSBM frequency was similar between groups after matching (Table 2). Weekly CSBMs increased from baseline in both groups (both *P* < 0.05), with a greater increase and a higher post-treatment frequency in the PFMS combined with BF-ES group (*P* < 0.05). The weekly CSBM responder rate was also higher with combined treatment than with BF-ES alone (91.5% vs. 80.0%; *χ*^2^ = 7.090, *P* = 0.008). Both groups improved in all sEMG measures (all *P* < 0.05; Table 3). Compared with BF-ES alone, PFMS combined with BF-ES produced larger improvements in coefficient of variation of the resting baseline, relaxation time after rapid contraction, and endurance-contraction variability (all *P* < 0.05). Post-treatment mean resting-baseline sEMG did not differ between groups (*P* > 0.05).

**Table 2:** Comparison of weekly CSBM frequency and responder rates.

Group	*N*	Weekly CSBM frequency before treatment, median (IQR), times/week	Weekly CSBM frequency after treatment, median (IQR), times/week	Weekly CSBM responder rate, *n* (%)	*χ*^2^ value	*P* value
BF-ES group	130	2 (2–3)	4 (3–5)^a^	104 (80.0)	7.090	0.008
PFMS Plus BF-ES group	130	2 (1–3)	4 (4–6)^a^^,^^b^	119 (91.5)		

a *P* < 0.05 vs. baseline in the same group.

b *P* < 0.05 vs. the BF-ES group after treatment.

**Table 3:** Comparison of sEMG parameters.

Group	Time	Mean resting baseline (μV)	Coefficient of variation of anterior resting baseline	Relaxation time after rapid contraction (s)	Coefficient of variation of endurance contraction
BF-ES group	Baseline	2.16 (1.05–4.06)	0.99 (0.89–1.29)	1.27 (1.24–1.32)	1.04 (1.03–1.09)
	8 weeks	1.51 (0.98–2.56)^a^	0.29 (0.28–0.33)^a^	0.46 (0.36–0.48)^a^	0.18 (0.13–0.23)^a^
PFMS Plus BF-ES group	Baseline	2.39 (1.21–3.43)	1.02 (0.96–1.22)	1.32 (1.23–1.35)	1.04 (1.03–1.06)
	8 weeks	1.41 (0.91–2.38)^a^	0.17 (0.16–0.23)^a^^,^^b^	0.33 (0.22–0.33)^a^^,^^b^	0.12 (0.11–0.14)^a^^,^^b^

a *P* < 0.05 vs. baseline in the same group.

b *P* < 0.05 vs. the BF-ES group after treatment.

### 3.3 Comparison of secondary efficacy outcomes between the two groups

After treatment, both groups demonstrated significant improvements in weekly laxative-use days, CSS, TCM constipation syndrome score and PAC-QOL scores compared with baseline (all *P* < 0.05; Table 4). Compared with the BF-ES group, the PFMS plus BF-ES group achieved greater reductions in weekly laxative-use days, TCM constipation syndrome score and PAC-QOL scores. No significant between-group difference was observed in post-treatment CSS scores (*P* > 0.05).

**Table 4:** Comparison of secondary efficacy outcomes.

Group	Time	Mean weekly days of laxative use (days)	CSS score (points)	TCM constipation syndrome score (points)	PAC-QOL scores (points)
BF-ES group	Baseline	5 (4–6)	12 (10–15)	23 (15–39)	2.29 (2.02–2.55)
	8 weeks	3 (1–3)^a^	6 (4–8)^a^	11 (6–19)^a^	1.25 (1.11–1.43)^a^
PFMS Plus BF-ES group	Baseline	5 (5–6)	12 (10–14)	35 (26–39)	2.29 (2.25–2.40)
	8 weeks	2 (0–3)^a^^,^^b^	6 (5–8)^a^	10 (6–12)^a^^,^^b^	1.18 (1.07–1.32)^a^^,^^b^

a *P* < 0.05 vs. baseline in the same group.

b *P* < 0.05 vs. the BF-ES group after treatment.

### 3.4 Comparison of clinical efficacy between the two groups

The overall response rate was higher in the PFMS combined with BF-ES group than in the BF-ES-alone group (97.69% vs. 82.31%, *P* < 0.05; Table 5). Analysis of treatment outcomes showed that a larger proportion of patients achieved markedly improved outcomes in the PFMS combined with BF-ES group (43.08% vs. 15.38%, *P* < 0.01). Meanwhile, the proportion of non-responders was lower in the PFMS combined with BF-ES group (2.31% vs. 17.69%, *P* < 0.01).

**Table 5:** Comparison of clinical efficacy grades.

Group	*N*	Non-responders [*n* (%)]	Improved [*n* (%)]	Markedly improved [*n* (%)]	Cured [*n* (%)]	Overall response rate [*n* (%)]
BF-ES group	130	23 (17.69）	87 (66.92）	20 (15.38）	0 (0)	107 (82.31）
PFMS Plus BF-ES group	130	3 (2.31)	70 (53.85)	56 (43.08)	1 (0.77)	127 (97.69）

### 3.5 Multivariable linear regression analyses of changes in clinical scores

No problematic multicollinearity was identified among the covariates included in the multivariable models; VIFs ranged from 1.066 to 2.801 and all tolerance values were >0.35. Change scores were calculated as post-treatment minus baseline values; therefore, more negative values indicated greater improvement. Separate multivariable linear regression models were performed for changes in CSS, TCM constipation syndrome, and PAC-QOL scores (Table 6). The model for CSS score change was not statistically significant overall (adjusted *R*^2^ = 0.019, *F* [10,249] = 1.508, *P* = 0.137), and treatment regimen was not independently associated with CSS score change (*β* = 0.028, SE = 0.403, *P* = 0.945).

**Table 6:** Multivariable linear regression analyses of changes in clinical scores.

Variable	Change in CSS score		TCM constipation syndrome score		Change in PAC-QOL score	
	*β* ± SE	*P* value	*β* ± SE	*P* value	*β* ± SE	*P* value
Age (years)	0.013 ± 0.014	0.379	0.032 ± 0.035	0.364	−0.002 ± 0.002	0.346
Disease duration (years)	−0.074 ± 0.030	0.014	0.107 ± 0.074	0.152	−0.004 ± 0.004	0.332
Weekly days of laxative use before treatment (days)	−0.050 ± 0.153	0.745	−0.128 ± 0.379	0.735	0.015 ± 0.020	0.469
Weekly days of laxative use during treatment (days)	−0.287 ± 0.206	0.165	−0.073 ± 0.513	0.886	−0.005 ± 0.027	0.863
Sex (male = 0, female = 1)	0.400 ± 0.532	0.453	−1.872 ± 1.321	0.158	0.148 ± 0.070	0.035
Structural anorectal abnormality (no = 0, yes = 1)	−0.542 ± 0.683	0.428	−1.805 ± 1.695	0.288	−0.016 ± 0.090	0.862
Pelvic-floor dyssynergia (no = 0, yes = 1)	−0.482 ± 0.624	0.441	−1.737 ± 1.550	0.263	−0.088 ± 0.082	0.286
Treatment regimen (PFMS plus BF-ES vs. BF-ES)	0.028 ± 0.403	0.945	−8.468 ± 1.001	<0.001	−0.203 ± 0.053	<0.001
Laxative use before treatment (no = 0, yes = 1)	2.690 ± 1.229	0.03	−11.238 ± 3.050	<0.001	0.252 ± 0.162	0.121
Laxative use during treatment (no = 0, yes = 1)	0.276 ± 0.734	0.707	10.569 ± 1.823	<0.001	−0.163 ± 0.097	0.093

The model for TCM constipation syndrome score change was statistically significant (adjusted *R*^2^ = 0.436, *F* [10,249] = 21.050, *P* < 0.001). After adjustment for the other covariates, PFMS plus BF-ES was associated with a greater reduction in TCM syndrome score than BF-ES alone (*β* = −8.468, SE = 1.001, *P* < 0.001). Laxative use before treatment was also associated with a greater reduction in TCM syndrome score (*β* = −11.238, SE = 3.050, *P* < 0.001), whereas laxative use during treatment was associated with a smaller reduction (*β* = 10.569, SE = 1.823, *P* < 0.001).

The model for change in PAC-QOL score was also statistically significant (adjusted *R*^2^ = 0.078, *F* [10,249] = 3.193, *P* < 0.001). PFMS plus BF-ES was associated with a greater reduction in PAC-QOL score (*β* = −0.203, SE = 0.053, *P* < 0.001). Female sex was associated with a smaller reduction in PAC-QOL score than male sex (*β* = 0.148, SE = 0.070, *P* = 0.035). Neither structural anorectal abnormality nor pelvic-floor dyssynergia was independently associated with changes in any of the three clinical scores.

### 3.6 Multivariable ordinal logistic regression analysis of TCM syndrome therapeutic efficacy grades in patients with ODS

Multivariable ordinal logistic regression showed that treatment regimen was independently associated with clinical efficacy grade (Table 7). After adjustment for age, disease duration, weekly laxative-use days before and during treatment, sex, structural anorectal abnormality, pelvic-floor dyssynergia, and laxative use before and during treatment, patients receiving PFMS plus BF-ES had greater odds of achieving a higher efficacy grade than those receiving BF-ES alone (*β* = 1.347, OR = 3.847, 95% CI 2.151–6.881, *P* < 0.001). Laxative use during treatment was associated with lower odds of achieving a higher efficacy grade (*β* = −1.674, OR = 0.187, 95% CI 0.069–0.506, *P* < 0.001). No significant independent associations were observed for structural anorectal abnormality or pelvic-floor dyssynergia. The overall model was statistically significant (likelihood-ratio *χ*^2^[10] = 78.988, *P* < 0.001). The test of parallel lines was not statistically significant (*χ*^2^[20] = 24.616, *P* = 0.217), indicating no evidence of violation of the proportional-odds assumption.

**Table 7:** Multivariable ordinal logistic regression analysis of clinical efficacy grades.

Variable	Coding	*β*	SE	Wald	OR (95% CI)	*P* value
Age (years)	Per 1-year increase	−0.002	0.01	0.03	0.998 (0.979–1.018)	0.863
Disease duration (years)	Per 1-year increase	0.02	0.021	0.901	1.021 (0.979–1.064)	0.342
Weekly days of laxative use before treatment (days)	Per 1-day/week increase	0.157	0.104	2.258	1.170 (0.953–1.436)	0.133
Weekly days of laxative use during treatment (days)	Per 1-day/week increase	−0.142	0.139	1.047	0.867 (0.660–1.139)	0.306
Sex	0 = male; 1 = female	0.403	0.366	1.214	1.496 (0.731–3.065)	0.27
Structural anorectal abnormality	0 = no; 1 = yes	0.208	0.46	0.205	1.232 (0.500–3.033)	0.65
Pelvic-floor dyssynergia	0 = no; 1 = yes	0.094	0.424	0.049	1.099 (0.479–2.523)	0.824
Treatment regimen	0 = BF-ES; 1 = PFMS plus BF-ES	1.347	0.297	20.629	3.847 (2.151–6.881)	<0.001
Laxative use before treatment	0 = no; 1 = yes	1.246	0.863	2.081	3.475 (0.640–18.878)	0.149
Laxative use during treatment	0 = no; 1 = yes	−1.674	0.507	10.92	0.187 (0.069–0.506)	<0.001

Model-estimated probabilities were consistent with the ordinal regression results (Supplementary Table S2). The predicted probability of non-response was lower with PFMS plus BF-ES than with BF-ES alone (3.86% vs. 16.42%), whereas the predicted probability of marked improvement was higher (44.49% vs. 14.28%).

### 3.7 Adverse events

No serious adverse events were observed. Mild, self-limiting discomfort occurred in 4 patients receiving PFMS combined with BF-ES (3.1%), including perineal or perianal discomfort, abdominal distension, and defecatory urgency. One patient receiving BF-ES alone (0.8%) experienced transient buttock or thigh twitching. All events resolved without intervention, and no local skin or systemic reactions were reported.

## 4 Discussion

In this propensity score–matched cohort of 260 patients, adding PFMS to BF-ES was associated with additional short-term clinical benefit. Compared with BF-ES alone, combined treatment was associated with a higher weekly CSBM responder rate, higher post-treatment weekly CSBM frequency, fewer weekly laxative-use days, and greater reductions in TCM constipation syndrome and PAC-QOL scores. In the multivariable analyses, PFMS plus BF-ES remained independently associated with greater reductions in TCM syndrome and PAC-QOL scores and with higher odds of achieving a better clinical efficacy grade. In contrast, treatment regimen was not independently associated with CSS score change. Several pelvic-floor sEMG parameters also improved more favorably with combined treatment, providing supportive, although indirect, evidence of improved pelvic-floor neuromuscular function.

Biofeedback provides real-time visual information on anorectal and pelvic-floor muscle activity, enabling patients to improve abdominal propulsive effort and learn coordinated relaxation of the pelvic floor and anal sphincter during attempted defecation (23). In the present BF-ES protocol, neuromuscular electrical stimulation was followed by sEMG-guided biofeedback training. Electrical stimulation may facilitate pelvic-floor muscle activation and sensory awareness, while visual feedback enables patients to practice voluntary contraction and relaxation according to predefined targets. Randomized trials summarized in evidence-based guidelines have demonstrated the efficacy of biofeedback for functional defecatory disorders, particularly dyssynergic defecation (24, 25). In addition, Lv et al. reported that electrical stimulation combined with biofeedback improved pelvic-floor function and patient-reported distress after reconstructive surgery (26). These findings provide a clinically plausible basis for the symptomatic and quality-of-life improvements observed in both treatment groups.

The greater improvement observed with combined treatment suggests that PFMS may provide incremental benefit when added to BF-ES. A recent randomized controlled trial in patients with rectocele-associated ODS found that magnetic stimulation combined with biofeedback produced greater improvements in pelvic floor muscle recruitment, anorectal function, and constipation-related quality of life than conventional biofeedback alone (11). PFMS may complement BF-ES through a different mode of neuromuscular stimulation: PFMS provides externally induced pelvic-floor activation and may enhance muscle recruitment and endurance, whereas BF-ES reinforces voluntary muscle control and defecatory coordination through sEMG-guided training. The more favorable sEMG changes observed with combined treatment are consistent with this complementary-mechanism hypothesis. Importantly, structural anorectal abnormalities and pelvic-floor dyssynergia were similarly distributed between the matched groups and were not independently associated with the main treatment outcomes in the adjusted models, suggesting that the observed treatment association was not explained by the simple presence or absence of these characteristics. However, phenotype-specific treatment effects cannot be determined because the severity of structural abnormalities and detailed anorectal physiological changes were not evaluated longitudinally.

The pattern of between-group differences was more consistent for CSBM-based outcomes and PAC-QOL than for the CSS score. CSBM directly reflects spontaneous bowel movements accompanied by a sense of complete evacuation and therefore captures a central functional goal of treatment. PAC-QOL assesses the broader patient-perceived burden of constipation and may therefore reflect clinically meaningful changes beyond bowel frequency alone. In contrast, the CSS combines several dimensions, including bowel movement frequency, straining, incomplete evacuation, time spent on defecation, need for assistance, failed evacuation attempts, and duration of constipation (19). Some components, particularly the duration-of-constipation item, would not be expected to change over an 8-week treatment period, whereas other components may improve to different extents. This may reduce the responsiveness of the total CSS score to short-term between-group differences. Previous studies have also shown that improvements in constipation symptoms, patient-reported outcomes, and anorectal physiological measures do not necessarily occur in parallel (27, 28). Nevertheless, the absence of a significant difference in CSS change should be interpreted cautiously rather than attributed solely to scale characteristics. The CSS regression model also had very low explanatory power, indicating that the measured covariates explained little of the variability in CSS change.

The PFMS plus BF-ES group showed greater improvements than the BF-ES group in three pelvic-floor sEMG parameters: the coefficient of variation of the resting baseline, relaxation time following rapid contraction, and the coefficient of variation during endurance contraction (all *P* < 0.05; Table 3). These changes may indicate more stable pelvic-floor muscle activity and better control of contraction and relaxation. One possible explanation is that PFMS enhances neuromuscular recruitment and activation, while BF-ES helps patients improve voluntary muscle control and defecatory coordination. This interpretation remains tentative because sEMG does not directly measure anorectal pressure or defecatory mechanics, and post-treatment anorectal manometry, balloon expulsion testing, and defecography were not available.

The multivariable analyses showed that the associations varied across clinical outcomes. PFMS plus BF-ES was associated with greater improvements in TCM syndrome and PAC-QOL scores, whereas no independent association was observed for CSS change. In the ordinal regression analysis, combined treatment was also associated with substantially greater odds of achieving a higher clinical efficacy grade, consistent with the higher overall response observed in the PFMS plus BF-ES group. The covariate findings also deserve some consideration. Women showed a smaller reduction in the overall PAC-QOL score than men, but this should be viewed as an exploratory finding rather than evidence of a direct sex effect. PAC-QOL reflects several aspects of constipation-related quality of life, including physical and psychosocial discomfort, worries and concerns, and treatment satisfaction, and the observed difference may not have arisen from bowel symptoms alone. Because domain-level PAC-QOL data were not consistently available in the retrospective records, we could not determine which component contributed most to this difference. Factors such as obstetric history, previous pelvic-floor surgery, or menopausal status may also have influenced quality-of-life responses but were not captured in the present dataset.

Laxative use appeared to reflect different clinical states before and during treatment. Patients using laxatives before treatment may have had a greater baseline symptom burden and therefore more room for improvement, whereas continued use during treatment may have marked persistent symptoms or the need for rescue medication. These associations are therefore better interpreted as indicators of disease burden and treatment response than as evidence that laxatives themselves altered treatment efficacy. Structural anorectal abnormalities and pelvic-floor dyssynergia were not independently associated with changes in CSS, TCM syndrome, or PAC-QOL scores, or with clinical efficacy grade. This finding does not lessen their clinical relevance. In the present analysis, a mild rectocele and a more pronounced intussusception were both captured simply as “structural abnormality present,” and pelvic-floor dyssynergia was likewise treated as a binary characteristic. Such broad categories may have obscured differences related to the type and severity of structural disease, the degree of functional impairment, or the coexistence of structural and functional abnormalities, all of which may influence how individual patients respond to PFMS-based treatment.

Both treatment regimens appeared to be generally well tolerated, and no clear short-term safety signal was identified in the available medical records. However, retrospective ascertainment and the short follow-up period may have resulted in under-recording of mild, uncommon, or delayed adverse events.

This study has several limitations. Its single-center retrospective design may have introduced selection bias and limits the generalizability of the findings. Treatment was selected in routine clinical practice rather than randomly assigned, based on clinical and anorectal assessment, suitability for PFMS, and patient preference. Although propensity score matching and multivariable adjustment were performed, residual confounding and causal uncertainty remain. Additionally, outcome assessors were not blinded to treatment allocation. Because several outcomes relied on patient-reported information and sEMG assessments, detection bias cannot be excluded. Although complete clinical records were required for inclusion, no patient who otherwise met the eligibility criteria was excluded solely because of incomplete records. Nevertheless, reliance on routinely collected clinical records may still introduce information bias related to documentation quality. Disease duration and laxative-use history were extracted from medical records and may have been incompletely documented or misclassified, while potentially relevant factors such as obstetric history and hormonal status were unavailable. Structural anorectal abnormalities were included in the analysis, but detailed measures of anatomical severity, including rectocele size, grade of rectal prolapse or intussusception, and degree of pelvic-floor descent, were not consistently available. sEMG was the main physiological measure assessed longitudinally and provides only an indirect measure of pelvic-floor function; without post-treatment anorectal manometry, balloon expulsion testing, or defecography, the proposed physiological mechanisms could not be directly confirmed. In addition, the TCM constipation syndrome score was developed within a traditional Chinese medicine framework, which may limit its interpretability and generalizability outside this clinical setting. PFMS was delivered within a predefined clinical parameter range, with intensity adjusted according to individual tolerance, introducing treatment heterogeneity within the combined-treatment group, and the additional 20 min of PFMS per session may have introduced confounding related to unequal treatment exposure and attention. Finally, the short follow-up period precluded evaluation of the durability of treatment effects and long-term safety.

## 5 Conclusion

In this propensity score–matched retrospective cohort, PFMS plus BF-ES was associated with greater short-term improvement than BF-ES alone in patients with ODS. Given the non-randomized treatment allocation, these findings should be considered hypothesis-generating and require confirmation in prospective randomized controlled trials.

## Data Availability

The data analyzed in this study is subject to the following licenses/restrictions: the data are available from the corresponding author upon reasonable request, subject to patient privacy and institutional data confidentiality restrictions. Requests to access these datasets should be directed to Ming Li, liming@ahtcm.edu.cn.
