# Supplementary material for: Pelvic floor magnetic stimulation combined with biofeedback-based electrical stimulation for obstructed defecation syndrome: a retrospective cohort study
Source: Front Med (Lausanne). 2026 Oct 29;13:1982655. doi: 10.3389/fmed.2026.1982655 (PMC13754691; doi:10.3389/fmed.2026.1982655)
Supplement: Supplementary file 1 [file Table1.docx]

| Supplementary Table1: Traditional Chinese Medicine (TCM) syndrome score for constipation | | | | | | | | |
| --- | --- | --- | --- | --- | --- | --- | --- | --- |
| Part A. Main symptoms (0/2/4/6) | | | | | | | | |
| Main symptom item | None (0) | | Mild (2) | | Moderate (4) | | Severe (6) | |
| Pain during defecation | 0 | | 2 | | 4 | | 6 | |
| Perianal heaviness/tenesmus | 0 | | 2 | | 4 | | 6 | |
| Insufficient urge to defecate (weak urge) | 0 | | 2 | | 4 | | 6 | |
| Weak straining on toilet | 0 | | 2 | | 4 | | 6 | |
| Fatigue after defecation | 0 | | 2 | | 4 | | 6 | |
| Part B. Secondary symptoms (0/1/2/3) | | | | | | | |  |
| Secondary symptom item | | None (0) | | Mild (1) | | Moderate (2) | Severe (3) |  |
| Mental fatigue | | 0 | | 1 | | 2 | 3 |  |
| Fatigue | | 0 | | 1 | | 2 | 3 |  |
| Shortness of breath | | 0 | | 1 | | 2 | 3 |  |
| Reluctance to speak | | 0 | | 1 | | 2 | 3 |  |
| Frequent sighing | | 0 | | 1 | | 2 | 3 |  |
| Irritability | | 0 | | 1 | | 2 | 3 |  |
| Depressed mood | | 0 | | 1 | | 2 | 3 |  |
| Abdominal distension/fullness | | 0 | | 1 | | 2 | 3 |  |
| Abdominal pain | | 0 | | 1 | | 2 | 3 |  |
| Epigastric stuffiness/fullness | | 0 | | 1 | | 2 | 3 |  |
| Belching | | 0 | | 1 | | 2 | 3 |  |
| Clear and profuse urination | | 0 | | 1 | | 2 | 3 |  |
| Short/red urination | | 0 | | 1 | | 2 | 3 |  |
| Night sweats | | 0 | | 1 | | 2 | 3 |  |
| Dizziness | | 0 | | 1 | | 2 | 3 |  |
| Cold pain in epigastrium/abdomen | | 0 | | 1 | | 2 | 3 |  |
| Soreness/weakness of waist and knees | | 0 | | 1 | | 2 | 3 |  |
| Five-center heat | | 0 | | 1 | | 2 | 3 |  |
| Insomnia with excessive dreaming | | 0 | | 1 | | 2 | 3 |  |
| Aversion to cold | | 0 | | 1 | | 2 | 3 |  |
| Cold limbs | | 0 | | 1 | | 2 | 3 |  |
| Thirst for warm drinks | | 0 | | 1 | | 2 | 3 |  |
| Dry mouth with scanty fluids | | 0 | | 1 | | 2 | 3 |  |
| Bitter mouth/halitosis | | 0 | | 1 | | 2 | 3 |  |
| Foreign-body sensation in throat | | 0 | | 1 | | 2 | 3 |  |
| Poor appetite | | 0 | | 1 | | 2 | 3 |  |
| Palpitations/anxiety | | 0 | | 1 | | 2 | 3 |  |
| Tinnitus | | 0 | | 1 | | 2 | 3 |  |
