# Supplementary material for: Pelvic floor magnetic stimulation combined with biofeedback-based electrical stimulation for obstructed defecation syndrome: a retrospective cohort study
Source: Front Med (Lausanne). 2026 Oct 29;13:1982655. doi: 10.3389/fmed.2026.1982655 (PMC13754691; doi:10.3389/fmed.2026.1982655)
Supplement: Supplementary file 2 [file Table2.docx]

Table S2 Model-estimated predicted probabilities of clinical efficacy grades by treatment arm

| Clinical efficacy grade | BF-ES | PFMS plus BF-ES |
| --- | --- | --- |
| Non-responder | 16.42% | 3.86% |
| Improved | 69.21% | 50.99% |
| Markedly improved | 14.28% | 44.49% |
| Cured | 0.09% | 0.66% |

Footnotes (important):

1. Values are the mean of individual-level predicted probabilities saved from the fitted ordinal logistic (proportional-odds) model.
2. Efficacy grade was coded as 0 = non-responder, 1 = improved, 2 = markedly improved, 3 = cured (higher values indicate better efficacy).
3. Predicted probabilities were computed from the multivariable model reported in Table 7; complete-case analysis was used (n=260).
